# Neuro-sensitization in cats with chronic pain and its association to osteoarthritis progression

**DOI:** 10.3389/fpain.2026.1723172

**Published:** 2026-05-26

**Authors:** Manuela Lefort-Holguin, Aliénor Delsart, Aude Castel, Colombe Otis, Maxim Moreau, Maude Barbeau-Grégoire, Bertrand Lussier, Jean-Pierre Pelletier, Johanne Martel-Pelletier, Eric Troncy

**Affiliations:** 1 Groupe de Recherche en Pharmacologie Animale du Québec (GREPAQ), Université de Montréal, Saint-Hyacinthe, QC, Canada; 2 Department of Clinical Sciences, Faculty of Veterinary Medicine, Université de Montréal, Saint-Hyacinthe, QC, Canada; 3 Osteoarthritis Research Unit, University of Montreal Hospital Research Center (CRCHUM), Montréal, QC, Canada

**Keywords:** age, cat, chronic pain, degenerative joint disease, nervous sensitization, pain severity

## Abstract

**Introduction:**

Feline osteoarthritis (OA) is characterized by somatosensory neuro-sensitization, assessed through quantitative sensory testing. It was hypothesized that somatosensory neuro-sensitization would increase with OA pain severity, as categorized through the validated Montreal Instrument for Cat Arthritis Testing, for Veterinarians (MI-CAT(V)).

**Methods:**

Healthy (*n* = 10) and cats with naturally occurring OA (*n* = 121) were enrolled in this prospective, negatively controlled study. Peripheral and spinal sensitization were respectively assessed by paw withdrawal threshold (PWT) and response to mechanical temporal summation (RMTS). PWT determined allodynia threshold. Derived from MI-CAT(V), cats were sorted into four validated OA severity clusters, from absent to severe OA pain. Outcomes were compared across allodynia status (healthy, non-allodynic and allodynic) and OA clusters, while testing for the influence of demographic data, with alpha set at 5%.

**Results and discussion:**

The PWT, RMTS, MI-CAT(V) outcomes and age accurately discerned between healthy and OA animals (*P* < 0.002), but not body weight. Non-allodynic cats had similarly altered MI-CAT(V) and RMTS to allodynic cats, but they were younger (*P* = 0.010) and had a higher PWT than allodynic (*P* < 0.001), and similar PWT (*P* = 0.925) but older (*P* < 0.001) than healthy cats. Spinal sensitization was similar in the three OA-affected clusters (mild-moderate-severe; *P* = 1.000), but MI-CAT(V) categorized them sensitively (*P* < 0.001). The mild cluster included more non-allodynic cats than the moderate (*P* = 0.021) and severe (*P* = 0.026) clusters. Interestingly, 32% of mild OA cats were allodynic, when the proportion increased to 61% in pooled moderate/severe OA cats (*P* = 0.013). OA cats are sensitized compared to healthy cats, and peripheral sensitization seems to increase with OA severity (or *vice-versa*?), which influences pain phenotype.

## 1 Introduction

Feline osteoarthritis (OA) is a complex condition that not only affects joint structure and mobility but also induces maladaptive changes in the nervous system, leading to chronic nociplastic pain (1). As feline OA is a degenerative pathology, the impacts on the animal's quality of life may vary according to the disease's state of progression.

The Groupe de recherche en pharmacologie animale du Québec (GREPAQ) demonstrated that the pain-related behavioral signs, assessed using a validated clinical metrology instrument—the Montreal Instrument for Cat Arthritis Testing, for use by Veterinarians (MI-CAT(V)) (2–5)—are linked to distinct biomechanical and activity profiles, reflecting the instrument's discriminatory abilities (5, 6). Validated objective outcomes measures showed that cats with severe OA pain presented higher lameness values, and more fatigue related to OA pain than cats with mild to moderate OA, according to the podobarometric gait analysis (PGA) and the stair assay compliance (SAC), respectively: mild, moderate > severe OA for PGA and SAC. Further, mildly affected cats were more active than moderately and severely OA-affected cats (mild > moderate, severe OA pain) according to nighttime actimetry monitoring (NAM) (5, 6). Collectively, these findings indicate that the MI-CAT(V) clusters enable the identification of different pain phenotypes.

Understanding the different pain phenotypes associated with feline OA may help predict a patient's response to a specific treatment. Indeed, Delsart et al. found that mildly OA-affected cats were less responsive to firocoxib treatment, a non-steroidal anti-inflammatory drug (NSAID), than moderately and severely affected cats (5). More importantly, they also experienced negative effects of NSAID withdrawal during the recovery period, whereas moderate and severe OA cats experienced residual analgesic effects (5). Considering these results, there is a need to determine personalized treatment plans that will suit each patient's needs and limit the associated side effects. Establishing the relationship between nervous sensitization and clinical pain signs (*i.e.,* daily functional alterations) will bring more insight into the different pain phenotypes, which may help veterinarians refine diagnosis and therapeutic planning in feline OA.

Quantitative sensory testing (QST) has previously shown promise in evaluating both somatosensory sensitization and changes in facilitatory and inhibitory pain pathways (7). Guillot et al. developed two QST outcomes for assessing feline OA neuro-sensitization: static QST with the paw withdrawal threshold (PWT) (8) and dynamic QST with the response to mechanical temporal summation (RMTS) (9). These allowed characterization of peripheral (secondary allodynia) and centralized (spinal wind-up) sensitization caused by chronic feline OA, respectively. According to the 2020 systematic review and meta-analysis on QST in feline OA published by Monteiro et al., 1) both PWT and RMTS outcomes were reliable (*i.e.,* repeatable and reproducible) and specific (*i.e.,* able to discriminate between healthy and OA affected cats) 2) OA cats showed strong evidence of peripheral and central sensitization in this metanalysis (7). However, the neuro-sensitization profile was not linked to pain expression in previous studies, particularly allodynia and hyperalgesia, or OA pain severity.

This study aimed to characterize the somatosensory profiles of cats with naturally occurring OA using validated QST outcomes and to determine how nervous sensitization relates to OA severity, and their association with demographic data. It was hypothesized that peripheral and central sensitization would increase in prevalence and intensity with greater functional impairment as defined by MI-CAT(V) scores (5), and that OA allodynic cats would present greater MI-CAT(V) score and aggravated central sensitization, compared to OA non-allodynic cats and healthy cats.

## 2 Methods

### 2.1 Ethical approval

The study was approved by the Institutional Animal Care and Use Committee of ArthroLab, Inc. (A196-ART22F) and of Université de Montréal—Faculty of veterinary medicine (CEUA-23-Rech-2029). Management of animals adhered to the regulations outlined in the Canadian Council on Animal Care, Guide to the Care and Use of Experimental Animals, Vol. 1 (1993, revised in Feb. 2017) and complied with the US Dept. of Agriculture Animal Welfare Act (9 CFR Parts 1–3). All procedures also adhered to the ARRIVE guidelines for reporting animal research and the Committee for Research Ethical Issues of the International Association for the Study of Pain guidelines.

### 2.2 Study design, inclusion criteria and animal housing

A total of 131 cats (*n* = 10 healthy, *n* = 121 OA) from the ArthroLab colony were enrolled in a comparative, blinded, controlled study. The inclusion criteria were related to physical examination findings—including body weight (BW), body condition score (BCS), health observations—clinical pathological findings (complete blood count, serum chemistries, and urinalysis), behavior, group sociability and acclimation performance. Cats with chronic kidney disease up to and including International Renal Interest Society stage 1 were eligible to enroll, provided the disease was stable. All selected OA cats (≥7 years old) were free of clinically significant abnormalities, except for naturally occurring OA in appendicular joints diagnosed with radiographic evidence and scored, as previously described (8, 10–12). Healthy cats had no preexisting condition, with no radiographic evidence of OA (10). All cats were neutered. Demographic data included age, sex, BW, BCS and radiographic (X-R) score. The cats were group-housed in rooms with controlled lighting, temperature, and humidity. Fresh tap water was available *ad libitum*, and food was provided twice daily (morning and afternoon), following the manufacturer's recommendations. Cats were also previously acclimated to the following outcomes evaluation: PWT, RMTS and MI-CAT(V). Measurements were conducted in absence of any recent exposure to analgesics, and a washout period of 16 weeks minimum was applied.

### 2.3 Quantitative sensory testing (QST)

Two validated QST outcomes were used to evaluate secondary allodynia (peripheral sensitization) and the spinal wind-up phenomenon (central sensitization): the PWT and RMTS, respectively (8, 9).

The front and hind limbs PWT were measured with an electronic von Frey esthesiometer (Rigid Tip 0.7 mm^2^ of surface 28G, IITC Life Science, Woodland Hills, CA, USA) fitted on a hand-held force transducer. The tip was placed perpendicularly into the plantar/palmar surface of the four paws while the cat was standing up in a meshed cage specifically mounted for this evaluation. The stimulus was gradually increased until the cat withdrew or elevated the paw, or displayed other avoidance behaviors such as vocalization, agitation, or jumping. The peak of force was recorded in grams. Each paw was tested once per session, and each session included four PWT measurements conducted in duplicate (separated by a 4 min interval) in a randomized order to prevent habituation or anticipation. Measurements below 2 g were considered unreliable and discarded. A cut-off of 200 grams was imposed to prevent injury. To identify highly sensitized cats, an allodynia threshold for either front limb or hind limb was established according to PWT data distribution of healthy and OA cats. Individuals with at least two values (on a minimum of four to a maximum of eight measurements) below the first quartile of all PWT data were considered allodynic cats (1, 8).

The spinal windup was quantified with the RMTS device (Top Cat®, Bespoke Measurement Systems, Cambs, UK). The RMTS device allowed to administer repetitive mechanical stimuli of sub-threshold intensity (one stimulus does not elicit a pain behavior) at predetermined intensity (4 N), duration (1.3 s.) and time intervals (0.385 Hz) (1, 7, 9). A round-ended metallic pin (2.5 mm diameter, 10 mm length) mounted on a light rolling diaphragm actuator delivered the mechanical stimuli. The stimulator was secured within a band placed around the limb, positioned on the cranial aspect of either the right or left mid-antebrachium, with a “dummy” device placed on the contralateral limb for control. During testing, cats were free to move within a meshed cage. The stimulation set was stopped by the evaluator when an obvious discomfort behavior (*e.g*., vocalization, agitation, striking the band containing the stimulator, or avoidance behavior) was detected, or stopped automatically when the maximal number of stimuli (30) was reached. The number of tolerated stimuli was recorded, and each assessment was conducted in duplicate, with a 4 min interval between trials during which the cats received positive reinforcement (affection and treats) to ensure the environment remained enriching and stress-free. A reliability score from 0 to 2 was assessed for each trial (0, not reliable; 1, fairly reliable; 2, reliable) to obtain a weighted average.

### 2.4 Montreal instrument for Cat arthritis testing, for veterinarians (MI-CAT(V))

The MI-CAT(V) evaluates body posture, gait, performance at two obstacles—one where the cat must pass through a trap door set at elbow height and the other where the cat must jump down an obstacle—all 15 items being scored 0 to 3, and ends with a numerical rating scale of 1 to 10 to establish a global assessment of the cat's OA condition (4). Blinded assessors (M.L.-H., M.Sc. candidate, A.DE., *Ph.D.* candidate, and Pr. E.TR.) independently conducted the evaluations, remaining unaware of the cat condition. The MI-CAT(V) score functions as an alteration index (expressed in percentage), where higher values indicate greater impairment in mobility and physical function associated to OA pain. The total score of all completed assessments is divided by 55. In case of no attempt for the obstacles, the maximal value, 4, is attributed but the maximal score remains 55 to overweight the no attempt in the percentage of alterations. Cats were sorted into validated OA severity clusters: absent (<9%), mild (9%–20%), moderate (21%–35%) and severe (>35%) OA using the MI-CAT(V) scores in line with its recently validated discriminatory ability (5, 6).

### 2.5 Statistical analysis

The statistical analyses were performed using SPSS software (IBM Corp. released 2011, IBM SPSS Statistics for Windows, version 20.0, Armonk, NY, USA). To validate outcome specificity, one-sided Mann–Whitney *U*-test was used to compare healthy, and OA affected cats, in agreement with the *a priori* hypothesis that neuro-sensitization would affect OA, and not healthy cats. Demographic data were compared across OA status, allodynia status and OA severity clusters. Pearson's chi-squared test was used (two-sided) to analyze categorical data such as observed sex frequencies. All outcomes were compared across allodynia status (healthy, non-allodynic and allodynic) and OA clusters using Kruskal–Wallis test with Bonferroni correction. To compare prevalence of allodynia across mild, moderate and severe OA cats, a two-sided Pearson Chi-Square Test was carried out, followed by Fisher test for individual comparison, when necessary. The analyses were performed with alpha value set at 5%. Complementary statistical analyses (on potential association with age) are presented in Supplementary material.

## 3 Results

There were no significant differences in BW (*P* = 0.696) and BCS (*P* = 0.075), or sex repartition (*P* = 0.22) between healthy and OA animals (Table 1), but this was different for both age and X-R score (*P* < 0.001). The three outcomes, PWT, RMTS and MI-CAT(V), accurately discerned between healthy and OA animals (*P* < 0.002) (Table 2).

**Table 1:** Mean (SD) results for demographics of the different OA severity clusters as assessed through the MI-CAT(V) scores.

OA severity cluster	Age (year)	Sex	BW (kg)	BCS (/9)	X-R score
Healthy (<9%) *n* = 10	2.5 (0.0)^a^	2F/8M	4.6 (0.7)	5.0 (0.2)	0.0 (0.0)^a^
Mild (9–20%) *n* = 22	8.7 (2.3)^b^	13F/9M	4.7 (0.8)	5.4 (1.1)	9.5 (4.3)^b^
Moderate (21–35%) *n* = 58	10.8 (2.2)^c^	23F/35M	5.0 (1.0)	5.2 (1.1)	8.7 (4.6)^b^
Severe (>35%) *n* = 41	11.0 (2.3)^c^	24F/17M	4.7 (0.8)	5.6 (2.1)	10.0 (5.7)^b^

BCS, body condition score; BW, body weight; F, female; M, male; MI-CAT(V), Montreal Instrument for Cat Arthritis Testing, for Veterinarians; OA, osteoarthritis; X-R, x-rays.

^a,b,c^ Different letters indicate a significant between-groups difference (per column). Please, refer to text for exact *P*-values.

**Table 2:** Mean (SD) results for the front and hind limb PWT, the RMTS and the MI-CAT(V) across OA severity clusters and allodynia status.

OA severity cluster	Allodynia status	Front limb PWT (g)	Hind limb PWT (g)	RMTS (count)	MI-CAT(V) score (%)
Healthy (<9%) *n* = 10	Healthy	175.0 (23.6)^a^	169.9 (22.8)^a^	25 (3)^a^	5.1 (2.7)^a^
Mild (9–20%) *n* = 22	Non-Allodynic *n* = 15	153.5 (36.0)^a^	161.8 (33.0)^a,b^	13 (7)^b^	16.9 (2.8)^b^
	Allodynic *n* = 7 (32%)	97.1 (32.9)^b^	130.1 (20.9)^b,c^	15 (10)^b^	15.8 (3.3)^b^
Moderate (21–35%) *n* = 58	Non-Allodynic *n* = 23	131.6 (33.4)^b^	148.4 (43.8)^b^	11 (6)^b^	28.3 (3.7)^c^
	Allodynic *n* = 35 (60%)	81.6 (40.3)^c^	104.8 (38.5)^c^	13 (7)^b^	27.9 (4.4)^c^
Severe (>35%) *n* = 41	Non-Allodynic *n* = 16	134.2 (43.6)^b^	159.0 (34.4)^b^	14 (7)^b^	40.9 (5.3)^d^
	Allodynic *n* = 25 (61%)	80.2 (39.0)^c^	107.5 (36.6)^c^	11 (5)^b^	40.8 (5.9)^d^

MI-CAT(V), Montreal Instrument for Cat Arthritis Testing, for Veterinarians; OA, osteoarthritis; PWT, paw withdrawal threshold; RMTS, response to mechanical temporal summation.

^a,b,c,d^ Different letters indicate a significant between-groups difference (per column). Please, refer to text for exact *P*-values.

Non-allodynic cats had similar front and hind limb PWT to healthy cats (*P* = 0.925), and allodynic cats had lower PWT than both healthy (*P* < 0.001) and non-allodynic cats (*P* < 0.001) tested for the OA group, as a whole. Contrary to our hypothesis of research, non-allodynic and allodynic cats had similarly decreased RMTS counts (*P* = 1.000) and similarly increased MI-CAT(V) scores (*P* = 0.487), both showing more altered outcome values compared to healthy cats (*P* < 0.001), again tested for the OA group, as a whole. The neuro-sensitization, as assessed through the allodynia status, worsened with age: healthy cats [2.5 (0.0) years] were significantly younger than non-allodynic [9.6 (2.6) years, *P* < 0.001] and allodynic cats [11.2 (2.0) years, *P* < 0.001], and allodynic cats were significantly older than non-allodynic cats (*P* = 0.010). However, BW, BCS and X-R score did not show any further distinction between non-allodynic and allodynic cats (*P* = 1.000).

**Figure 1:**
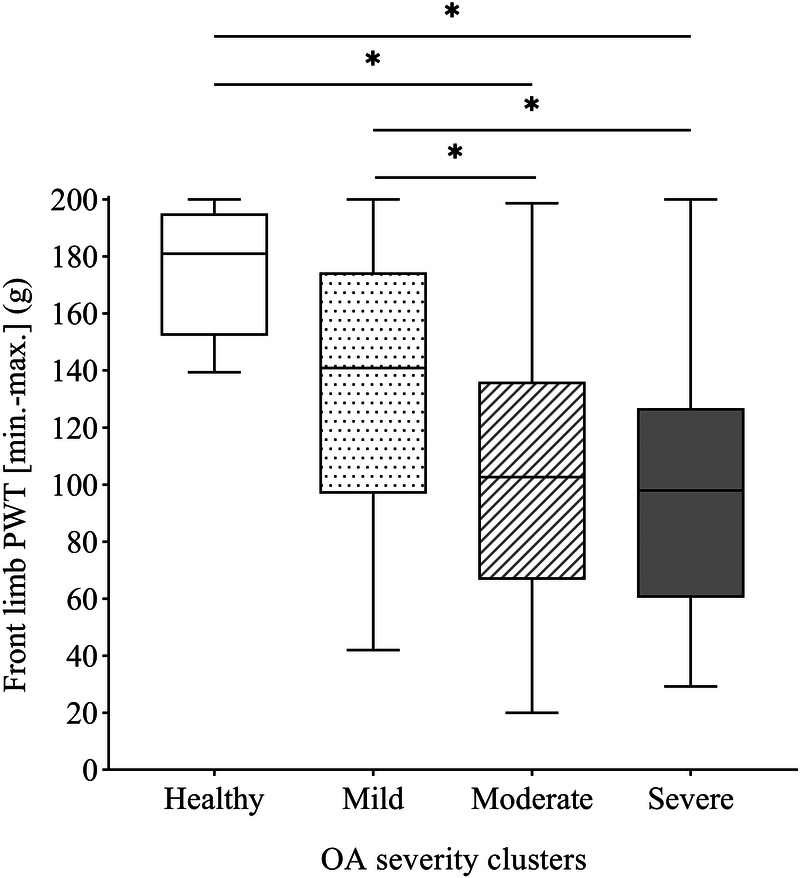
Front limb paw withdrawal threshold (PWT) across osteoarthritis (OA) severity clusters (mild, 9%–20%; moderate, 21%–35%; and severe, >35%) based on the Montreal instrument for Cat arthritis testing, for veterinarians [MI-CAT(V)]. The upper and lower limits of the box plots represent the 75th and 25th percentiles, respectively. The horizontal bars at the ends of the lines outside the boxes represent the minimal and maximal measures. The horizontal line within the box plots represents the median value. Moderate and severe OA cats presented a significantly decreased front limb PWT *vs*. both mild OA and healthy cats. *Significant difference for inter-clusters comparison after application of Bonferroni correction. Please, refer to text for exact *P*-values.

The *n* = 131 cats were sorted into the four OA severity clusters, each being represented and considered in the analysis: absent (*n* = 10), mild (*n* = 22) moderate (*n* = 58) and severe OA (*n* = 41). Moderately and severely OA-affected cats had a significantly lower front limb PWT than healthy cats (both *P* = 0.008) and mildly affected cats (moderate OA, *P* = 0.033; severe OA, *P* = 0.036). No differences in PWT were found between healthy and mildly affected cats (*P* = 0.656) as well as between moderately and severely affected cats (*P* = 1.000) (Figure 1). Similar results were found with hind limb PWT as moderately and severely affected cats had a significantly lower hind limb PWT than healthy cats (moderate OA, *P* = 0.009; severe OA, *P* = 0.026). However, significant differences were not reached when compared to mildly OA-affected cats (moderate OA, *P* = 0.055; severe OA *P* = 0.181).

The three OA-affected clusters showed similar centralized sensitization (*P* = 1.000) and significantly lower RMTS counts than healthy cats (mild OA, *P* = 0.001, moderate OA, *P* < 0.001; severe OA, *P* < 0.001). The MI-CAT(V) score across OA severity analysis categorized the clusters specifically (*P* < 0.001), but the difference was not statistically significant between the absent OA *vs.* mild OA clusters. Age also seemed to increase according to OA severity: absent [2.5 (0.0) years] OA cluster < mild [8.7 (2.3) years, *P* = 0.033] < moderate [10.8 (2.2) years] and severe [11.0 (2.3) years] (both *P* < 0.001 *vs*. absent OA; and *P* = 0.013 for moderate, *P* = 0.012 for severe, respectively *vs*. mild) OA clusters. Other demographic criteria (*i.e.*, sex, BW, BCS and X-R score) had no influence between the mild, moderate and severe OA clusters (*P* = 1.000, except sex frequencies *P* = 0.684) (Table 1).

**Figure 2:**
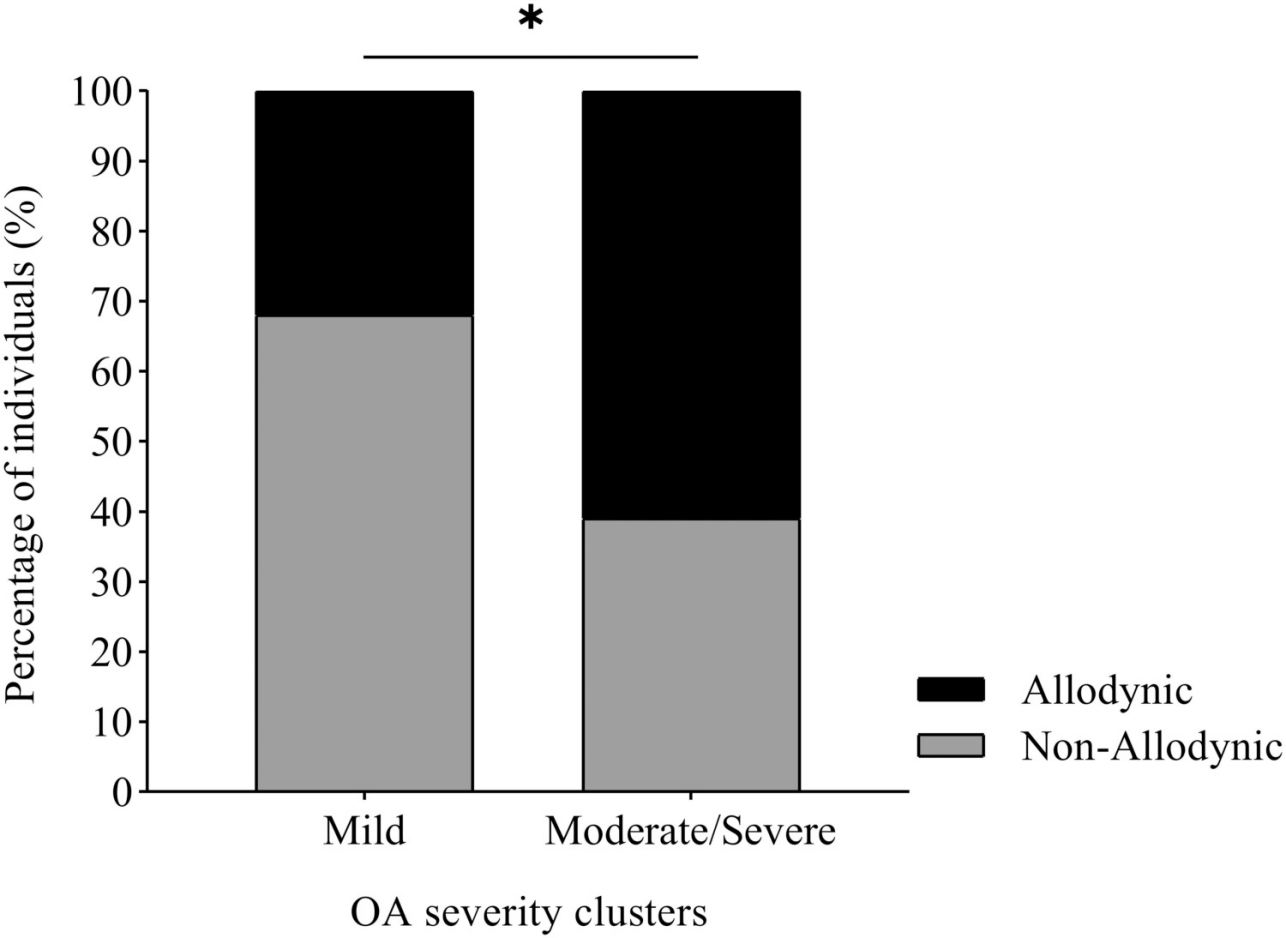
Distribution of cats (in %) according to allodynia status (allodynic *vs*. non-allodynic) and osteoarthritis (OA) severity cluster (mild, 9%–20%; and moderate/severe, >20%) based on the Montreal Instrument for Cat Arthritis Testing, for Veterinarians [MI-CAT(V)]. Mild OA includes 68% of cats as non-allodynics, whereas 61% of moderate/severe OA were allodynic. *P = 0.013 for inter-clusters comparison.

As for the prevalence of allodynia across the OA severity clusters (Figure 2), 15-of-22 (68%) mildly affected cats were non-allodynic when 35-of-58 (60%) moderately affected cats and 25-of-41 (61%) severely affected cats were allodynic (*P* = 0.049). There were significantly more non-allodynic cats in the mild cluster than in the moderate (*P* = 0.021) and severe (*P* = 0.026) clusters. Upon these results, 32% of mild OA cats were allodynic, and the proportion increased to 61% in the pooled moderate/severe OA cluster (*P* = 0.013), in accordance with our research hypothesis.

## 4 Discussion

In this controlled study, the neurobiological dimension of feline OA was explored to characterize the relationship between the somatosensory neuro-sensitization and the severity of OA pain clinical symptoms. Previous results suggested that pain phenotype—based on the validated MI-CAT(V) pain scale—can provide a more complete understanding of the feline OA phenotype and justify more targeted treatment strategies (5, 6).

The present findings demonstrate a link between somatosensory neuro-sensitization and OA pain severity, especially in peripheral nociceptive pathways. Cats in the moderate and severe OA clusters showed evidence of peripheral sensitization through lowered PWT, compared to healthy and mild OA cats. Furthermore, it was found that only 32% of OA cats with mild functional alterations were allodynic when 61% of OA cats with moderate to severe functional alterations showed signs of peripheral hypersensitization. The OA pain severity cluster analysis did not bring further distinction on central sensitization between mildly, moderately and severely OA-affected cats, the three clusters showing similar evidence of spinal sensitization according to their RMTS counts. The absence of change in RMTS counts between OA severity levels may reflect an early plateau in central sensitization development or that the RMTS device is lacking sensitivity to detect differences in spinal sensitization.

This study also allowed for further outcome validation as the PWT, RMTS and MI-CAT(V) score were sensitive and specific to the somatosensory condition, with OA cats having sensitized somatosensory system and higher functional alterations, compared to healthy cats. Moreover, OA cats were older and presented higher structural alterations than healthy cats. Interestingly, BW/BCS was not a risk factor to current OA in our population sample. This point was suspected in a recent narrative review, suspecting the claimed influence of BW/BCS in feline OA to be over-extrapolated from other species (13), and would need to be confirmed with a larger epidemiological study.

The allodynia status analysis did not differentiate spinal sensitization nor functional alterations, as allodynic and non-allodynic cats had similarly altered RMTS counts and MI-CAT(V) scores. Neither BW/BCS, nor X-R score was predictive of allodynia [and OA severity], suggesting the overweight or the structural alterations to be not linked to neuro-sensitization [and functional alterations]. However, it looks like allodynia development required more time as the allodynic cats [11.2 (2.0) years] were significantly older than the non-allodynic cat [9.6 (2.6) years]. Comparing MI-CAT(V) scores across OA severity brought further validation to the established clusters except for the absent and mild OA clusters. This was likely a Type II statistical error, as the sample size for healthy and mild OA cats were smaller. Nevertheless, there was evidence of a linear aggravation in MI-CAT(V) score between OA severity clusters.

Allodynic cats had lower PWT compared to healthy and non-allodynic cats, thus validating the methodology and confirming stratification of peripheral sensitization. Lastly, this study identified age as a risk factor for aggravation of neuro-sensitization [and OA severity], as the incidence of allodynia [and MI-CAT(V) score] significantly increased with age. This suggests that neuro-sensitization and chronic nociplastic pain develops progressively over time in the physiopathology of feline OA. As recently proposed in a scoping review on translational pain neuro-sensitization (1), the hypothesis of a counteracting balance between facilitatory and inhibitory nociceptive endogenous controls is associated with chronic pain in feline OA up to an imbalance favoring the facilitation and a rupture point where the fatigue of the inhibitory endogenous controls leads to a hypersensitized status.

Feline OA is increasingly recognized as more than just a degenerative joint disease; growing evidence highlights the involvement of the nervous system. Chronic OA pain triggers complex biochemical and physiological responses, including immune-inflammatory activation, neuroplastic changes, heightened neural excitability, and disruptions in the balance between pain facilitation and the body's natural inhibitory controls (1, 7). These processes contribute to nervous system sensitization, which alters how pain is perceived in the brain's somatosensory regions (1), and the condition, in absence of intervention, will aggravate with time. This form of chronic maladaptive pain is particularly challenging to diagnose and treat in OA cats, generating significant consequences for their overall health and welfare (1).

Research specifically addressing the treatment of somatosensory neuro-sensitization in feline OA remains limited. To date, only five peer-reviewed studies have evaluated the impact of pharmacological —including meloxicam, tramadol, and gabapentin (4, 8, 11, 12) — and nutritional (therapeutic diet enriched in omegas-3, turmeric extract and hydrolyzed collagen) (14) interventions on peripheral and/or central sensitization in cats with naturally occurring OA. Given the relevance of neuro-sensitization mechanisms in chronic pain physiopathology, understanding the efficacy of these treatments is essential. The following section reviews existing evidence (Table 3), emphasizing the importance of pain phenotype stratification in the interpretation of treatment responses.

**Table 3:** Therapeutic efficacy for feline OA pain management.

Therapy	PGA	SAC	AM	MI-CAT(V)/MI-CAT(C)	PWT	RMTS	References
Meloxicam	+	?	+	?/+	o	o	(8, 11, 18, 24, 25)
Firocoxib	+	+	+	+	+/o^a^	+/o^a^	(5, 42)
Tramadol	+	?	+	?	?	+	(12)
Gabapentin	?	?	+/−	?	+	?	(4, 32)
Frunevetmab	?	?	+/o	+ (trend)^a^	+^a^	+^a^	(43, 44)
Therapeutic diet^b^	+	+	+	+	+	+ (trend)^a^	(6, 14)

o, no effect; +, positive effect (improvement); −, negative effect (degradation);?, effect unknown; AM, actimetry monitoring; MI-CAT(C), Montreal Instrument for Cat Arthritis Testing, for Caretakers; MI-CAT(V), Montreal Instrument for Cat Arthritis Testing, for Veterinarians; OA, osteoarthritis; PGA, podobarometric gait analysis; PWT, paw withdrawal threshold; RMTS, response to mechanical temporal summation; SAC, stairs assay compliance.

a GREPAQ internal data.

b Therapeutic diet enriched in marine source-omega-3s, hydrolyzed collagen and turmeric extract. (trend) denoted a clinically significant change and the lack of statistical significance was associated to a type-II statistical error (*P* < 0.010).

NSAIDs are currently the first-line pharmacological option for managing feline OA. According to the 2024 ISFM and AAFP consensus guidelines, meloxicam (Metacam®, Boehringer Ingelheim) and robenacoxib (Onsior®, Elanco) are the only NSAIDs recommended for long-term use in cats (15). While a few studies have investigated the clinical efficacy of meloxicam (non-selective COX inhibitor) in OA management (*n* = 13 (4, 8, 11, 16–25)), only one study [*n* = 1 (26)] has evaluated robenacoxib (COX2 selective inhibitor, or coxib) specifically in this context and more importantly, only two studies have examined their effects on nervous system sensitization following treatment durations of three to four weeks (8, 11).

Guillot et al. assessed the effects of various meloxicam doses (0.025 mg/kg, 0.04 mg/kg or 0.05 mg/kg PO, daily) on the NAM, PGA, and PWT of cats with naturally occurring OA. While improvements in locomotor activity (NAM) were observed, the results on the PGA were inconclusive and it was found that meloxicam failed to improve allodynia thresholds, indicating a lack of effect on peripheral sensitization (8). It was suggested that meloxicam treatment efficacy was likely influenced by the distribution of neuro-sensitized cats within the treatment groups (8). The group receiving daily 0.04 mg/kg had the highest rate of allodynic cats and exhibited overall reduced responsiveness to treatment (8). In contrast, the other groups with lower rates of allodynic cats receiving either 0.025 mg/kg or 0.05 mg/kg showed more favorable responses in both locomotor activity and kinetic outcomes (8). These findings suggest that allodynic cats experienced persistent pain despite the NSAID treatment, whereas non-allodynic cats benefited more from it, displaying increased mobility and comfort as well as improved limb function (8).

Monteiro et al. evaluated the effects of meloxicam (0.05 mg/kg PO, daily) on central sensitization using the RMTS and while treatment was associated with increased nighttime activity (*i.e.*, NAM) and improved weight-bearing (*i.e.*, PGA), RMTS values remained unchanged, indicating no reduction in spinal wind-up phenomenon (11). This suggests that meloxicam does not effectively target central sensitization mechanisms in this context (11). Nevertheless, coxibs are believed to cross the blood brain barrier as they are more lipophilic and less acidic than non-selective NSAIDs like meloxicam (27, 28). This could enable them to act on the central nervous system either by blocking the arachidonic cascade directly at the dorsal horn or by activating the descending pain inhibitory pathways (28–30). Such anti-neuro-sensitization effects were observed with firocoxib on both PWT and RMTS in OA cats, and specifically on non-allodynic cats (GREPAQ—internal data). While conventional NSAIDs remain a cornerstone in feline OA management, their limited efficacy on somatosensory neuro-sensitization and their known side effects underscore the need for adjunctive and alternative therapies.

Other drugs such as tramadol—an agonist to *μ*-opioid receptors and serotonin and norepinephrine reuptake inhibitor—have been observed to influence nervous sensitization. Monteiro et al. reported a positive effect of tramadol on the RMTS, suggesting that this drug could reverse spinal sensitization and improve biomechanics in OA cats (11, 12). In fact, tramadol (3 mg/kg PO every 12 h) had a significant treatment effect, over three weeks of administration, on spinal sensitization, actimetry and PGA (11, 12). In the same fashion, tramadol has been shown to be effective in reducing temporal summation and secondary allodynia/hyperalgesia in other species, including humans (for review, see Frezier et al., 2025) (1). Tramadol might be an interesting avenue for the management of OA, either alone or in combination with other therapeutic approaches. However, as only four articles have been published on the efficacy of tramadol for feline OA management (4, 11, 12, 31), further studies are warranted to better understand the general mechanism of tramadol in OA cats.

Gabapentin exerts its analgesic effects by binding to the *α*2*δ* subunit of N-type voltage-gated calcium channels located presynaptically. This interaction reduces calcium influx into neurons, thereby decreasing the release of excitatory neurotransmitters such as glutamate, norepinephrine, and substance *P* (32, 33). Mechanisms not directly related to neurotransmitter release at dorsal horn include inhibition of descending serotonergic facilitation, stimulation of descending inhibition anti-inflammatory actions, and influence on the affective component of pain (32). In feline OA, gabapentin has shown variable efficacy (4, 34). Administration at 10 mg/kg PO every 8 h for 30 days was associated with modest improvements in somatosensory neuro-sensitization and spontaneous locomotor activity in a small cohort of cats (4). However, a lower dosing regimen (10 mg/kg orally every 12 h for 14 days) failed to demonstrate significant analgesic effects and primarily induced sedation without meaningful pain relief (34).

Monoclonal antibodies targeting nerve growth factor represent a promising pharmacological avenue to address the underlying neurobiological mechanisms of chronic OA pain. By inhibiting nerve growth factor signaling, these agents aim to disrupt key pathways involved in nociceptor sensitization and chronic pain amplification (1, 30, 35, 36). Concurrently, nutraceutical-enriched therapeutic diets, particularly those rich in omega-3 polyunsaturated fatty acids, are also gaining attention as viable non-pharmacological option for modulating somatosensory neuro-sensitization in OA. The anti-inflammatory and neuroprotective properties of omega-3 fatty acids, including eicosapentaenoic acid, docosahexaenoic acid, and docosapentaenoic acid, are mediated through the production of specialized pro-resolving mediators such as resolvins, maresins and protectins (37, 38). These bioactive lipid mediators exert effects on both immune and glial support cells, contributing to the resolution of inflammation and attenuation of neuroinflammatory processes (37–41). Beneficial effects of a therapeutic diet enriched in omegas-3, turmeric extract and hydrolyzed collagen on peripheral and central sensitization of OA cats have been recently reported (14). Together, these novel approaches offer complementary strategies for addressing the multifactorial nature of OA pain, with the potential to improve clinical outcomes beyond conventional NSAID therapy.

### 4.1 Limitations

Study limitations include the imbalance in sample sizes across severity clusters, particularly the small size of the healthy and mildly affected groups, which may have affected statistical power. Similar limitation must exist about the inference to provide for age effect, as the selection of healthy cats could be biased on young cats to get null X-R score, as inclusion criterion. Therefore, the latter sample could be not representative of the healthy population. Other outcomes of neuro-sensitization could be assessed, such as electrodiagnosis or evaluation of pain signature cerebral integration through functional neuroimaging or electroencephalography (either evoked potentials, and/or spectral analysis). The observational nature of this study precludes causal inference. Moreover, the absence of treatment evaluation limits the interpretation of QST metrics as predictive tools for therapeutic responsiveness.

## 5 Conclusion

This study bridges neurophysiological and functional assessment in feline OA by demonstrating that peripheral sensitization increases with disease severity (or, *vice-versa*?). Both look to be positively linked to age, when BW/BCS appear to not be a risk factor in feline OA, and structural alterations (X-R score) to be not linked to neuro-sensitization or functional alterations. The use of QST outcomes alongside MI-CAT(V) offers a multidimensional view of pain phenotype, which could support personalized analgesic strategies. As it was shown in past publications, the severity of the observed functional alterations influences the pain phenotype, changing the kinetic and activity profiles (mild, moderate > severe OA, for PGA and SAC) (5, 6). According to the present study, moderately and severely OA-affected cats are more often allodynic, as shown by decreased PWT (secondary peripheral sensitization), compared to mildly affected cats (mild > moderate, severe OA, for PWT and NAM). The use of such pain phenotypes will allow veterinarians to target specific treatment plans that will attempt to simultaneously improve biomechanical alterations, increase locomotor activity and fight somatosensory neuro-sensitization associated to feline OA.

## Data Availability

The datasets presented in this study can be found in online repositories. The names of the repository/repositories and accession number(s) can be found below: https://data.mendeley.com/datasets/djzyzv75tn/1.
