# Supplementary material for: Neuro-sensitization in cats with chronic pain and its association to osteoarthritis progression
Source: Front Pain Res (Lausanne). 2026 May 26;7:1723172. doi: 10.3389/fpain.2026.1723172 (PMC13246664; doi:10.3389/fpain.2026.1723172)
Supplement: Supplementary file 1 [file Datasheet1.pdf]

Complementary statistical analyses:

At the Reviewer #4 request, we would like to indicate that power calculation was applied based on our previous analyses of neuro-sensitization in OA cats vs. healthy cats, as well as responsiveness to treatment of neuro-sensitization. Indeed, with such sample size of OA cats, the calculated power of analysis was very high (87.84%), so we did not find useful to include it in the manuscript. This used a Type I error rate of 5%, and the mean (SD) values in PWT and RMTS we collected previously.

Moreover, in these previous analyses, we did not stratify the OA cats based on their MI-CAT(V) assessed OA pain severity, therefore such power calculation was not adequate for the present analyses.

Finally, as we systematically presented the variability associated to all measurement by including mean (SD) in each outcome, we did not feel necessary, at this level of observational description, to calculate effect size. For the age analysis, we explain below the incapacity to conduct a covariate adjustment and the tests of association that were conducted.

A statistical analysis including age as a covariate could not be possible with regards to the collected data: These were one-measure for each cat, and not repeated measures over time. Each cat had PWT measured on both front and hind-limbs in duplicate. In that case, this represented eight measurements (duplicate of each paw). For some cats, a duplicate was not completed, and a single measurement on each paw was collected successfully.

For this reason, we tested for a potential association of age with the neuro-sensitization clusters (three groups of healthy, non-allodynic OA, and allodynic OA cats) or with the OA severity (assessed through MI-CAT(V) clusters: mild, moderate, severe) for each neuro-sensitization measure (**PWT-front**, **PWT-hind**, and **RMTS**). This was not conclusive (see below) meaning age to have no (or minimal) influence on neuro-sensitization [and OA severity] inside each cluster, but neuro-sensitization clusters (healthy, non-allodynic and allodynic) [and OA severity clusters] were sensitive to age, and not to BW, BCS and X-R score (structural alterations), as highlighted in the current version of the manuscript.

Therefore, this suggests **aging was associated with the neuro-sensitization worsening**, as determined by the three clusters, but inside each cluster, age had no (or minimal) influence. This was the same for the OA severity clusters. **Neuro-sensitization and OA pain severity aggravated with age, meaning the neuro-sensitization to aggravate with the OA pain severity, and *vice-versa***. Moreover, we agree this point to be crucial, as the Reviewer #1 highlighted it too, and we clearly emphasized its role in the Discussion: “Lastly, this study identified age as a risk factor for aggravation of neuro-sensitization [and OA severity], as the incidence of allodynia [and MI-CAT(V) score] significantly increased with age,” as well as in the Abstract and the Conclusion.

Association of age with neuro-sensitization [and OA severity] was tested inside each cluster using the non-parametric test of correlation, Rho of Spearman (Rho_S). It was either non-significant, or when significant (only for PWT-hind and all OA cats: Rho_S =-0.470, P = 0.008; as well as PWT-hind and OA non-allodynic cats: Rho_S = -0.424, P = 0.015; and PWT-hind in mild – Rho_S = -0.450, P = 0.020 –, moderate – Rho_S = -0.300, P = 0.012 –, and severe OA – Rho = -0.506, P < 0.001 –), the association was a **low-to-moderate** (< 0.51) level. It is comforting to observe that there is a **negative correlation between age and PWT-hind** in **OA cats** (*vs*. healthy) and in each OA severity cluster, but the association being not so strong, we hesitated to add this information as it will only repeat the yet presented correlations (with stronger association). For this reason, and in agreement with the Reviewer #4 suggestion, we added this information in Supplementary material.
